# Digital Identities and Verifiable Credentials

**DOI:** 10.1007/s12599-021-00722-y

**Published:** 2021-10-04

**Authors:** Johannes Sedlmeir, Reilly Smethurst, Alexander Rieger, Gilbert Fridgen

**Affiliations:** 1 Project Group Business and Information Systems Engineering of the Fraunhofer FIT, FIM Research Center, University of Bayreuth, Bayreuth, Germany; 2 SnT: Interdisciplinary Centre for Security, Reliability and Trust, University of Luxembourg, Luxembourg City, Luxembourg

**Keywords:** Blockchain, Digital wallet, European digital identity, Privacy, Public key infrastructure, Self-sovereign identity

## Introduction

Public institutions and companies typically employ physical credentials (such as passports, social security cards, and employee badges) to identify individuals. Individuals can choose where to store their physical credentials, and sometimes, they can decide to whom their credentials are disclosed. These familiar privileges inspired a new type of digital credential called a *verifiable credential* (VC). Similar to physical credentials, individuals can store their verifiable credentials in a so-called digital wallet on their mobile phone, on another edge device, or in the cloud, and they can use verifiable credentials for identification, authentication, and authorization (Sporny et al. 2019).

Verifiable credentials and digital wallets offer a convenient, secure, and privacy-oriented alternative to current physical and digital identity management systems. A recent example – COVID-19 vaccination certificates – highlights this. The verification of paper-based vaccination certificates is often error-prone and time-consuming, especially when many certificates have to be verified in a short period of time, e.g., at a football match or when boarding a plane. Moreover, to establish a sufficient level of authenticity, paper-based vaccination certificates are typically disclosed with additional personal information and identity documents, such as a physical ID card. This requirement to disclose a considerable amount of personal information raises privacy concerns, it is inconvenient, and it increases the total verification time.

The storage of vaccination-related digital information in a centralized database enables faster and more convenient verification, yet it also raises ethical, security, and privacy concerns. Such databases can facilitate unintended profiling, they are appealing targets for hackers, and they typically limit individuals’ control over the processing of their personal data (Rieger et al. 2021). The European Union thus permits Member States’ governments to directly issue EU Digital COVID Certificates to wallets that are controlled by citizens (European Commission 2021b). Although this development is notable, EU Digital COVID Certificates cannot yet be stored in a standardized wallet alongside a broad array of documents, certificates, and credentials that can be used to prove a subject’s identity (Rieger et al. 2021). Further work remains to be done.

This is precisely what motivates the development of verifiable credentials and standardized digital wallets. In this catchword, we introduce this decentralized, interoperable approach to digital identity management. In particular, we discuss the challenges of today’s centralized identity management and investigate current developments regarding verifiable credentials and digital wallets. Finally, we offer suggestions about promising areas of research into *decentralized digital identities*.

## Review of the Status Quo

### Basic Terms

To create a common basis for discussion, it is important to first define the terms *digital identity* and *subject*. A digital identity is often defined as a digital reference to a person (Alamillo-Domingo 2020). It is thus something that a subject has and uses in response to requests for digital identification, authentication, or proofs of authorization. A digital identity consists of attributes that can be revoked, deleted, transferred, or exchanged, such as citizenship, institutional affiliations, and proofs of ownership (Lyons et al. 2019; Preukschat and Reed 2021). Identity attributes are typically connected to a subject via a unique identifier within a system or domain, such as an index in a database or a social security number (Bosworth et al. 2005).

The subject of a digital identity is often a human, but the subject can also be a legal entity, an animal, or a device, among other things. Hence discussions of digital identities should not place an exclusive emphasis on human subjects (Dietz and Pernul 2020; Fedrecheski et al. 2020; Zwitter et al. 2020). Subjects can prove their identity attributes using credentials (Clauß and Köhntopp 2001). A credential can be a password that demonstrates ownership of a particular identifier like an email address, or else a credential can be a verifiable document issued by a third party that specifies identity attributes like a government-issued ID card (Bosworth et al. 2005).

### Identity Data Stored in Centralized Silos

In today’s web, digital identities are predominantly managed via service-specific user accounts that involve username-password combinations. Everyday complaints concerning the relationship between users and password-based authentication methods are numerous. Users struggle, for example, to manage the growing number of passwords (Novakouski 2013), and they can fall victim to password theft schemes such as phishing, key-logging, viruses, and malware (Herley 2009). Furthermore, users cannot seamlessly transfer identity-related information from one of their accounts to another. Users are thus required to repeat tedious registration processes, in which they disclose ID cards, a driver’s license, bank account information, and more. Federated identity management aims to mitigate some of these issues via Single Sign-On platforms that transfer identity-related information between services that are connected to the platform (Maler and Reed 2008).

Corporate digital identity platforms are consequently built upon large centralized silos that store identity data. These silos are a predominant cause of concerns due to security weaknesses, dubious data-sharing and surveillance ethics, and compromised privacy rights (Chaum 1985; Nofer et al. 2017; van der Aalst et al. 2019; Zuboff 2019; Nayar 2012). In August 2013, a successful hack compromised every one of Yahoo’s three billion user accounts (Oath 2017). Hacks of this nature are not just embarrassing; they are also costly (Cowley 2019; Preukschat and Reed 2021). In June 2014, Aleksandr Kogan, a researcher from Cambridge University’s Psychometrics Centre developed a personality quiz app for Facebook. Approximately 270,000 Facebook users installed the app and – unwittingly, in most cases – granted the app access to their identity data as well as their friends’ identity data. The app generated a private database that contained information about 50 million Facebook users. Aleksandr Kogan sold the database to a firm named Cambridge Analytica, who then used the database to construct 30 million psychological profiles about voters (Meyer 2018). Since the incident did not involve a hack, it is primarily an ethical problem (Aiello 2018). On 20 March 2018 the United States’ Federal Trade Commission opened an investigation to see if Facebook violated a data-sharing consent decree from 2012 (McKinnon 2018).

Government-managed digital identity platforms are affected by many of the same ethical and security problems as corporate digital identity platforms (Khera 2018; Ganesh 2018; Nayar 2012; Pahwa 2017). India’s national government, for example, created a digital identity platform named Aadhaar, which uses biometric data to reduce fraud and leakage. If biometric data is stolen or compromised, it is difficult to reverse the damage (Pandya 2019). One cannot ask a third party to reset one’s fingerprints, for instance, the way that one can request a password reset. Aadhaar’s centralized trust model notably failed to prevent an Aadhaar enrolment center’s supervisor from issuing an illegal ID for his dog (Jain 2015; Ganesh 2018). Problems also arise when Aadhaar shares citizens’ data with various service providers. In 2017, for example, the Kerala State government’s pension department copied information from the Aadhaar database and shared 3.5 million citizens’ data without the citizens’ consent. The citizens’ names, addresses, telephone numbers, bank account details, Aadhaar identifier-numbers, and photographs were published to the pension service’s website, which is visible to all (Tarafdar and Bose 2019). A year later, a security expert discovered a similar flaw: a State-owned service provider named Indane copied Aadhaar data and allowed potentially anyone to access information about citizens’ names, Aadhaar identifier-numbers, and bank account details (Christopher 2018; Graglia et al. 2018).

Government-managed digital identity systems nonetheless offer some notable benefits in comparison with physical identity management systems (World Bank 2015; Clark et al. 2019). Estonia’s and Slovenia’s national governments, for example, used digitization to reduce ID-related administration costs. Estonia’s national electronic identification document (eID) and X-Road data exchange layer saves an estimated two percent of Gross Domestic Product per year by reducing paper-based, ID-related transaction costs. Slovenia’s Ministry of Social Affairs generates savings via a digital platform that can verify identity information across more than 50 databases. Despite its flaws, the Aadhaar system eliminates many fake and duplicate beneficiaries of government programs, which results in significant savings (World Bank 2018b). Private companies in India also benefit from the Aadhaar system. Aadhaar reduced an Indian firm’s typical customer on-boarding cost from 1500 rupees to 10 rupees (World Bank 2018a).

## Decentralized Digital Identities

### Proposals for Change

Concerns about centralized digital identity platforms – managed either by companies or governments – are not new. In 2005, Microsoft’s Chief Identity Architect, Kim Cameron fretted, “If we do nothing, we will face rapidly proliferating episodes of theft and deception that will cumulatively erode public trust in the Internet”. Researchers began to focus on attribute-based access control systems that enable the authentication of natural persons based on a public key infrastructure (PKI) (Backes et al. 2005; Lioy et al. 2006). In spite of their technical advantages, these solutions failed to achieve mainstream adoption (Kubach et al. 2020; Novakouski 2013).

In 2013, Timothy Ruff and Jason Law founded Evernym in response to this “growing digital identity crisis” (Andrade-Walz 2019). Evernym’s staff later created the international, non-profit Sovrin Foundation and donated code to Linux’s Hyperledger Aries and Indy projects. Over the last few years, many similar projects have emerged in response to concerns about centralized platforms. Table 1 summarizes the broad array of identified problems and proposed, decentralized solutions.

**Table 1:** Various centralized problems and proposed, decentralized solutions

Problem diagnoses	Proposed solutions
Big Tech and government surveillance	Non-correlatable identifiers
“Big”, identity data silos	“Little”, distributed identity wallets
Digital identity theft and credential fraud	Machine-readable verifiable credentials
Hacked certificate authorities (centralized PKI)	DLT and KERI (decentralized PKI)
ID vendor lock-in and “walled gardens”	Open, interoperable, digital ID standards
Complicated password management	Password-free, PKI-based authentication
Specialized hardware for PKI-based authentication	User-friendly wallet apps on common devices
“Data creep” and excessive demands for attributes	Selective disclosure and consent management
Constant reliance on online identity providers	Offline, bi-lateral communication

Decentralized digital identity projects typically employ verifiable credentials and digital wallets. Their popularization can be traced to a 2017 proposal by Andrew Tobin and Drummond Reed from Evernym and Sovrin. According to the proposal, users of verifiable credentials and digital wallets do not have to purchase and carry specialized security hardware such as NFC smart-cards, encrypted USB wallets, or a Google Titan Security Key. Tobin and Reed’s proposal also does away with the need for identity data silos and password-based authentication, and it increases subjects’ control over the disclosure and exchange of their data. The scope of Tobin and Reed’s proposal is intentionally broader than the FIDO2 Project that focuses on password-free authentication; and since interoperability is crucial to Tobin and Reed’s proposal, it is also distinct from domain-specific login apps (Ehrlich et al. 2021; Tobin and Reed 2017).

In 2019, the World Wide Web Consortium (W3C) issued a formal Recommendation of verifiable credentials. The W3C defined verifiable credentials as digital documents issued with digital signatures. Asymmetric (public/private key) cryptography protects the digital signatures from corruption. For enhanced privacy, zero-knowledge proofs can additionally be used to reveal only the minimum of information required in an interaction. This feature is called *selective disclosure* or *minimal disclosure* (Sporny et al. 2019). Some information required by verifiable credentials is meant to be public, such as credential schemas or the revocation status of particular credentials (Tobin 2018). This public information can be stored on a blockchain like Hyperledger Indy or by a X.509 certificate authority (Chadwick 2020).

The storage of verifiable credentials in digital wallets is fundamentally different from the storage of individuals’ identity information in large, government-managed databases (like India’s Aadhaar) or in monetized data silos owned by Big Tech companies (like Alphabet, Amazon, Apple, Facebook, and Microsoft). Many decentralized identity projects also make use of *decentralized identifiers* (DIDs). DIDs are references to information about a subject’s public keys and associated metadata. DIDs enable end-to-end encrypted communication without the need for a third party. DIDs can be permanent and public, which is useful for public institutions. Alternatively, they can be temporary and private, which helps individuals resist third parties’ attempts to track their online interactions and correlate their identity information (Reed et al. 2021).

Cryptographic key pairs are also stored in digital wallets, together with verifiable credentials. Information derived from verifiable credentials can be released from a digital wallet in response to requests from various service providers, even if an Internet connection is not available (e.g., via Bluetooth or NFC). This requires secure and standardized, bi-lateral communication. The W3C is currently developing a new protocol, named DIDComm, for this purpose (Reed et al. 2021).

### Signs of Momentum and Political Tensions

Support for verifiable credentials and digital wallets is growing, especially in Europe and North America. There are a few notable examples. Canadian public authorities created the Verifiable Organizations Network (VON); the European Union established the European Self-Sovereign Identity Framework (ESSIF), which utilizes the European Blockchain Service Infrastructure (EBSI); Germany’s Federal Chancellery initiated a decentralized digital identity project; and the Linux Foundation established the Trust over IP Stacks.

Canada’s Verifiable Organizations Network uses verifiable credentials to issue digital licenses, permits, and registrations to legal entities (Bouma and Robert 2021). The network’s credentials can be stored in wallets that are compatible with Hyperledger Aries. The network is currently supported by the provincial governments of British Columbia and Ontario as well as the federal government of Canada (Jordan 2018). The European Blockchain Services Infrastructure uses verifiable credentials to issue official documents from public institutions, such as digital diplomas and social security passes (European Commission 2020). Germany’s Federal Chancellery initiated a decentralized digital identity project in 2021 that is based on verifiable credentials and Aries-compatible wallets. The Chancellery’s project uses verifiable credentials to issue a digital version of Germany’s physical eID card plus company travel documents, so that hotels in Germany can implement fast and secure, digital check-in processes without requiring a hardware eID card reader (Federal German Government 2021).

The Trust over IP Foundation seeks to align various decentralized digital identity projects. It issues global compatibility guidelines for Hyperledger Aries and Indy, verifiable credentials, and closely related technologies. Trust over IP was established in May 2020 by the Linux Foundation, following initial efforts from the provincial government of British Columbia, esatus, Evernym, IBM, Kiva, and Mastercard (Dizme et al. 2020). Trust over IP aims to compose “the digital trust layer that was missing in the original design of the Internet” – trust that is required for global, digital identification (Linux Foundation 2020).

As a counterpoint to the support for verifiable credentials and digital wallets, there is resistance from incumbents. A notable case is the W3C Verifiable Claims Task Force, which began work in November 2015. The following year, the Task Force proposed the formation of a W3C Verifiable Claims Working Group, which is able to issue an official Recommendation. (A Task Force is not.) Approval for the formation of a Working Group requires a vote of the W3C’s full membership. Many W3C members voted against the Verifiable Claims Working Group, so the proposal almost failed (Reed 2018). A public email, written by Michael Champion from Microsoft, highlighted the political nature of the dispute. Champion asserted that the W3C’s role is to observe industry developments, not to lead. According to him, the proposed Verifiable Claims Working Group could not “decree a solution that will succeed by force of W3C’s authority”. Champion acknowledged the similar arguments raised in public by Chris Wilson from Google and Tantek Çelik from the Mozilla Corporation (Champion 2016).

Mozilla reiterated its opposition in August 2020 when it argued that California’s Assembly Bill Number 2004, “Medical test results: verification credentials”, should be rejected, for it “dictates one particular technical approach” – the use of W3C verifiable credentials to communicate COVID-19 test results (Riley 2020). The Electronic Frontier Foundation and the American Civil Liberties Union also issued criticism of the bill. California’s Governor, Gavin Newsom, vetoed the bill on 25 September 2020. As for Microsoft, they later contributed to industry developments and launched version 1.0 of their Identity Overlay Network (ION) in March 2021. ION uses verifiable credentials and a blockchain-agnostic “sidetree” protocol as a PKI.

### Self-Sovereign Identity

**Table 2:** “SSI” is one name given to very different projects

Projects labelled “SSI”	Crypto-assets	Use of VCs	Aries-compatible wallet	Issuance fee-tokens	Use of blockchain	On-chain identifiers required	Use of ZKPs
Aries/Indy affiliates	No	Yes	Yes	No	Yes	No	Yes
ESSIF, ID_Alastria	No	Yes	No	No	Yes	Yes	No
Everest	Yes	Yes	No	Yes	Yes	Yes	No
Microsoft’s ION	No	Yes	No	No	Yes	Yes	No
InfoCert’s Dizme	No	Yes	Yes	Yes	Yes	No	Yes
MATTR	No	Yes	No	No	No	No	Yes
Ontology	Yes	Yes	No	Yes	Yes	Yes	Yes
Procivis’s eID+	No	No	No	No	No	No	No
Serto (ex-uPort)	Yes	Yes	No	No	Yes	Yes	No
Spruce’s Credible	No	Yes	No	No	Yes	No	No
Verifiable Credentials Ltd.	No	Yes	No	No	No	No	No
Workday	No	Yes	No	No	Yes	Yes	No

Self-Sovereign Identity (SSI) is a contested name that is often used to promote various decentralized digital identity projects (Preukschat and Reed 2021; Chadwick 2020; Cheesman 2020; Halpin 2020; Kubach et al. 2020). The Sovrin Foundation recently defined SSI as a set of community-sourced ethical principles that pertain to digital identities, privacy rights, and personal information (Sovrin Foundation 2020). SSI’s ethical principles typically assert that individuals should not cede a disproportionate amount of control to centralized digital identity providers like Big Tech companies or governments (Allen 2016; Spiekermann and Korunovska 2017).

Companies like esatus, Evernym, and Trinsic use the name SSI to market decentralized digital identity solutions that involve verifiable credentials, Aries-compatible wallets, and a blockchain-based PKI; but other companies use the same name to market solutions that do not necessarily involve any of these things (Gasteiger 2021; Kubach et al. 2020; Kuperberg 2020). Table 2 briefly illustrates SSI’s ambiguity and lack of commonality.

The European Blockchain Partnership, the European IDunion consortium, the Spanish Alastria Network, and Germany’s Federal Chancellery use the name SSI to promote citizens’ control over their identity data. Informal, web-based commentaries likewise associate SSI with individuals’ data property rights and privacy ethics (Reed and Sabadello 2019; Windley 2020; Preukschat and Reed 2021; Sabadello 2021). It is worth noting that, within formal contexts, sovereignty is typically discussed in relation to governance and State powers, not individuals’ identities or individuals’ property rights (Reijers et al. 2018; Foucault 1978).

SSI is sometimes associated with controversial politics and hyperbole (Graglia et al. 2018; Preukschat and Reed 2021; Windley 2020; Bouma and Robert 2021; Fry and Renieris 2020; Speelman 2020; Ishmaev 2020; Schneider 2019; Giannopoulou and Wang 2021; Cheesman 2020). In the United States, for instance, the political concept of self-sovereignty is embraced by “sovereign citizens” – individuals that refuse to acknowledge any government authority whatsoever (MacNab 2012; Ruff 2018; Haas 2016). Hence the following statement issued by Kim Cameron from Microsoft: “Self-Sovereign Identity makes me think of hillbillies on a survivalist kick” (Cameron 2018). The online Sovereign Individual movement also has an affinity with SSI. The movement significantly contributes to demand for decentralized digital identity solutions (Preukschat and Reed 2021). To understand what motivates the Sovereign Individual movement, Alex Preukschat and Drummond Reed from Evernym recommended a book called *The Sovereign Individual: How to Survive and Thrive During the Collapse of the Welfare State*. Preukschat and Reed (2021) describe the book’s authors as “prescient about the [online] decentralization movement”, even though the type of decentralization promoted by *The Sovereign Individual* is avowedly anti-democratic and “apocalyptic” (Davidson and Rees-Mogg 1997; O’Connell 2018). It is yet to be determined if SSI’s association with controversial politics and hyperbole will affect its adoption (Welling 2018; Bouma and Doerk 2020).

## Research Opportunities

Decentralized digital identity – based on verifiable credentials and standardized digital wallets – is a rapidly evolving topic. Its implications are especially relevant to incumbent services that rely on the collection of personal information and usage data. Decentralized digital identity presents multiple opportunities for information systems research. Table 3 lists potential avenues and exemplary research questions.

**Table 3:** A suggested research agenda for decentralized digital identities

Research avenue	Exemplary research questions
Applications of decentralized digital identities	When are decentralized digital identity systems justified?
	How can worthwhile applications for decentralized digital identities be classified?
Implications of decentralized digital identities	How will decentralized digital identities affect strategies and business models that are driven by user profiles?
	How will decentralized digital identities affect the management of business processes?
Decentralized digital identities and blockchain	How does the use of blockchain affect decentralized digital identity projects?
	How do decentralized digital identity projects influence the development of blockchain technologies?
Regulation of decentralized digital identities	How can decentralized digital identity systems balance privacy and transparency requirements?
	How will decentralized digital identities affect eGovernment services?
Governance of decentralized digital identity systems	How does the governance of decentralized digital identity systems differ from centralized systems?
	How can governance become aligned across different decentralized digital identity systems?
	How can governance frameworks accommodate machine-to-machine interactions?
Design choices for decentralized digital identity systems	How do different design choices affect the capabilities of decentralized digital identity systems?
	How do competing design choices affect the adoption of decentralized digital identities?
Socio-technical theories and decentralized digital identities	How does the association with SSI principles affect decentralized digital identity projects?
	How do legal frameworks and cultural values affect the adoption of decentralized digital identity systems?
	How do decentralized digital identities affect organizational practices?

A first avenue for research is the assessment of worthwhile applications for verifiable credentials and digital wallets. In general, verifiable credentials and digital wallets are appropriate if: (a) the fast, machine-verifiable exchange of identity-related information is desired without the direct interaction of issuers and verifiers, (b) centralized identity management systems present privacy and security concerns, and (c) centralized identity management systems fail to achieve adoption among a diverse array of stakeholders due to a lack of trust or a fear of concentrated market power. The latter topic is strikingly similar to established research about the adoption of blockchain technology (Pedersen et al. 2019). The application of digital identities should be considered beyond natural persons to also include organizations and smart devices (Fedrecheski et al. 2020).

Since decentralized digital identity management has the potential to affect business models that collect identity information and usage data, research can assess and investigate the consequences. How will companies that collect usage data adjust to the prospective adoption of decentralized digital identities? Can regulation prevent service providers from requesting more information from users than they require? How will decentralized digital identities affect data-driven platform strategies (De Reuver et al. 2018) and personalized advertisements (De Keyzer et al. 2015)?

Innovation in digital identity management is also important to consider when designing and managing business processes (Klarl et al. 2009; Mendling et al. 2020). Verifiable credentials and digital wallets can potentially disrupt e-commerce registration and on-boarding processes. An order on an e-commerce website could be completed, for example, by a user who has not previously registered with the website but who does have a digital wallet. The user could scan a single QR code to confirm the disclosure of identity information stored in verifiable credentials, such as their address, their age, or their credit card details. The European IDunion consortium explores these opportunities, with the aim to reduce customer lock-in effects that benefit large platforms like Amazon, Uber, and Airbnb.

A third promising avenue for research is the nexus of verifiable credentials, digital wallets, and blockchain technology. As noted, many decentralized digital identity projects use Hyperledger Indy or Ethereum-based blockchains like Hyperledger Besu to register information that needs to be publicly available. While a close association with blockchain may have helped the incubation of decentralized digital identity projects (Mühle et al. 2018), the long-term effects of this association are less clear. Hence we believe there are opportunities for research regarding the relationship between decentralized digital identity projects and blockchain development communities.

If blockchain is used with care and diligence, decentralized digital identity systems can ensure a high level of privacy. This is especially true if sensitive personal data is exchanged bi-laterally and selectively. A high level of privacy, however, introduces its own set of challenges, especially if privacy complicates the work of law enforcement authorities (Federal Office for Migration and Refugees 2021). Decentralized digital identity systems must therefore balance privacy and transparency requirements, which creates further opportunities for research, especially in the area of eGovernment services (Fridgen et al. 2018). Decentralized digital identity systems might allow citizens to better control the collection and exchange of their personal data by public authorities; but since public authorities in Europe and North America are typically bound by strict laws that regulate their data-processing activities, adding citizen consent as a mandatory second lawful basis may complicate cooperation and communication between authorities in certain cases (Federal Office for Migration and Refugees 2021).

Research to date has addressed the governance of blockchains more than the governance of decentralized digital identity systems. It remains to be seen how the governance of decentralized identity systems differs from today’s centralized alternatives, and how governance can be aligned between different systems and across national borders. We expect many similarities but also a few key differences when the governance of decentralized digital identity systems is compared to the governance of blockchain-based systems. Moreover, governance frameworks should incorporate digital identities for machines, since verifiable credentials can be used to identify and authenticate devices that belong to an individual or a business (Fedrecheski et al. 2020). Verifiable credentials can also be issued to sensors that feed data to smart contracts in order to authenticate the data and prove that the sensors were made by a trusted manufacturer. This may help address the “oracle problem” that is familiar to blockchain researchers (Swan 2015).

The consequences of different design options for decentralized digital identity systems are yet to be properly assessed. Such assessments should not only take into account the perspectives of participating organizations but also those of regulators and users. It is yet to be determined if the adoption incentives are sufficient for wallets that are designed to store only identity-related information. If not, then wallets might need to additionally store central bank digital currencies and/or crypto-assets. Other design-specific examples include different privacy options for verifiable credentials (Hardman 2019) as well as different resolution methods for decentralized identifiers (in combination with their corresponding PKI options) (Reed et al. 2021). Interesting research questions emerge from the competing design choices made by different projects. It remains to be seen, for example, if the German Federal Chancellery’s use of the Hyperledger Aries/Indy stack can be reconciled with the use of Hyperledger Besu by the European Blockchain Services Infrastructure and the Spanish Alastria Network.

Finally, there are multiple opportunities for socio-technical research into decentralized digital identity systems (Pinch and Bijker 1984; Sahay and Robey 1996; Bryant 2006). Socio-technical researchers can study, in particular, the effects of legal frameworks, cultural values, and privacy debates on the adoption and use of decentralized digital identity systems (Leidner and Kayworth 2006; O’Hara 2018; Fry and Renieris 2020); they can examine the different problem diagnoses that decentralized digital identity solutions are expected to address (Williams and Hummelbrunner 2010; Checkland and Poulter 2020); and they can explore the crucial relations between the various governance structures and technical designs (Zwitter et al. 2020). It is also worth examining if a proximity to SSI-related controversies affects decentralized digital identity projects (Ghent University 2020).

## Conclusion and Future Outlook

Verifiable credentials and standardized digital wallets offer a convenient, secure, and privacy-oriented alternative to both physical means of identification and centralized digital identity platforms. Governmental support for verifiable credentials and digital wallets is particularly strong in Canada and Germany, yet the future outlook is difficult to predict. To be successful, decentralized digital identity projects need to gain more traction and establish interoperability via a common governance framework (Wagner et al. 2020; Lundy 2019). What is required is “guidance within a legal architecture” (Fry and Renieris 2020). More specifically, verifiable credentials and blockchain-based PKI must be recognized as compliant with identity-related regulation, such as the European Union’s Electronic Identification, Authentication and Trust Services Regulation (Alamillo-Domingo 2020; The Council of the European Union 2014). The legally binding ID_Alastria model (developed in Spain), the German government’s support of several Hyperledger Aries/Indy-based projects, and the European Self-Sovereign Identity Framework are significant early steps. The next major steps will perhaps follow the European Commission’s recent announcement about European Digital Identity wallets (European Commission 2021a).

Decentralized digital identity management can expect to face continued resistance from incumbents. Some experts expect “wallet wars” not just for payments but also for digital identities, similar to the competition between browsers or mobile operating systems (Reed 2020). Apple, for example, recently announced their aim to integrate a wallet app that can store a digital driver’s license in the next version of their mobile operating system, iOS 15 (Business Insider 2021).

Research can play an important role in the prospective shift towards decentralized digital identities. Research is required to investigate the actual impact of decentralized digital identities on enterprises, individuals, and societies; it can help design suitable solutions; and it can determine if the adoption incentives for recent, decentralized digital identity solutions are superior to those of past, attribute-based PKI solutions.
